# A novel SHARPIN-PRMT5-H3R2me1 axis is essential for lung cancer cell invasion

**DOI:** 10.18632/oncotarget.18957

**Published:** 2017-07-04

**Authors:** Tingxiong Fu, Xiuwei Lv, Qingzhi Kong, Changjing Yuan

**Affiliations:** ^1^ Department of Pharmacy, The Central Hospital of Wuhan, Tongji Medical School, Huazhong University of Science and Technology, Wuhan, Hubei, China; ^2^ Department of Oncology of Integrated Traditional Chinese and Western Medicine, The Central Hospital of Wuhan, Tongji Medical School, Huazhong University of Science and Technology, Wuhan, Hubei, China; ^3^ The Central Hospital of Wuhan, Tongji Medical School, Huazhong University of Science and Technology, Wuhan, Hubei, China

**Keywords:** SHARPIN, PRMT5, cancer invasion, histone modification, gene expression

## Abstract

SHARPIN (Shank-associated RH domain interacting protein) is the main component of the linear ubiquitin chain activation complex (LUBAC). SHARPIN is involved in regulating inflammation and cancer progression. However, whether SHARPIN plays an important role in lung cancer metastasis and the potential underlying mechanism are still unknown. Here, for the first time, we reported that SHARPIN expression is closely related to lung cancer progression. Moreover, SHARPIN plays a central role in controlling lung cancer cell metastasis. Mechanistic studies further revealed that PRMT5 (Protein arginine methyltransferase 5), responsible for catalyzing arginine methylation on histones, is a novel cofactor of SHARPIN. This finding provides the basis for further study of the crosstalk between protein ubiquitination and histone methylation. We further found that SHARPIN-PRMT5 is essential for the monomethylation of histones of chromatins at key metastasis-related genes, defining a new mechanism regulating cancer invasion. A novel MLL complex (ASH2 and WDR5) was implied in the link between histone arginine2 monomethylation (H3R2me1) and histone lysine4 trimethylation (H3K4me3) for the activation of metastasis-related genes. These novel findings establish a new epigenetic paradigm in which SHARPIN-PRMT5 has distinct roles in orchestrating chromatin environments for cancer-related genes via integrating signaling between H3R2me1 and H3K4me3.

## INTRODUCTION

Lung cancer is one of the leading lethal cancers worldwide. However, the underlying mechanisms of this deadly cancer remain largely unknown [1, 2]. Better elucidation of the pathologic mechanisms involved in lung cancer metastasis will enable the development of better treatment options for patients.

SHARPIN (Shank-associated RH domain interacting protein) is the main component of the linear ubiquitin chain activation complex (LUBAC), which gives rise to linear-type ubiquitin chains via linkages between methionine 1 and glycine 76 in proteins [3, 4]. LUBAC has been found to add a linear polyubiquitin chain to IkB kinase (IKK) complex to activate nuclear factor-κB (NF-κB) signaling. Linear ubiquitination is also involved in the regulation of inflammation and immune signaling [5].

Recently, an increasing amount of evidence has demonstrated that SHARPIN is closely associated with oncogenesis. SHARPIN expression is frequently high in multiple human cancer types, including liver, ovarian, prostate, and breast cancer. SHARPIN plays tumor-associated roles during cancer biogenesis by promoting cell survival, growth, and invasion [6–10].

To explore the pathologic role of SHARPIN in regulating cancerogenesis, in the present study, we report for the first time that SHARPIN expression is upregulated in lung cancer. Moreover, we found PRMT5 as a novel cofactor of SHARPIN in regulating cancer-related gene expression. PRMT5 belongs to the protein arginine methyltransferase (PRMT) family, which transfers methyl groups from S-adenosylmethionine (SAM) to the guanidine nitrogen of arginine residues in proteins. PRMT5 is the main writer of symmetric dimethylarginine (Rme2s) and monomethylarginine (Rme1) [11–12]. Arginine methylation in histones is a common post-translational modification (PTM) of histones. Alterations in epigenetic regulation via the PTM of histones are highly correlated with cancer etiology and can lead to metastasis. Modulation the PTM of histones in chromatins is a key mechanism used by cancer cells to regulate transcription [13–14].

Here, we sought to determine how SHARPIN–PRMT5 interaction with its broader chromatin niche contributes to its ability to regulate cancer-related gene transcription. We demonstrate that the interaction with SHARPIN with a novel cofactor PRMTA5 plays biological roles in tumor progression and invasion via regulating a unique histone H3R2 methylation-coupled transcriptional activation in lung cancer cells.

## RESULTS

### SHARPIN controls invasive phenotype of lung cancer cells

**Figure 1:**
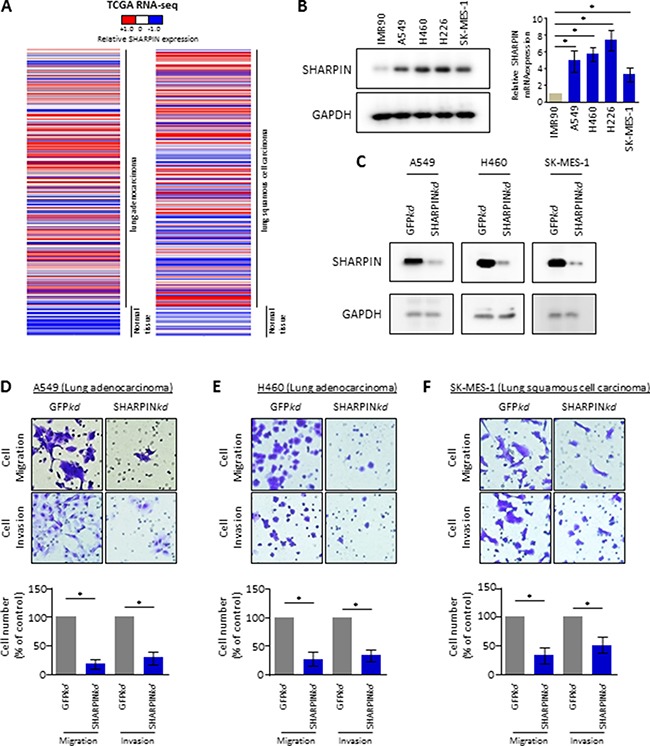
SHARPIN is a novel regulator of cell mobility in lung cancer cells (**A**) Heat map depicting gene expression of SHARPIN from RNA-Sequencing data with lung adenocarcinoma (LUAD, normal tissues vs tumor tissues) or squamous cell carcinoma (LUSC, normal tissues vs tumor tissues) in The Cancer Genome Atlas (TCGA). Expressions of SHARPIN are ranked by sample types. RNA-Seq values are shown by indicated bar. (**B**) SHARPIN expression profile in lung normal fibroblast cells and lung cancer cell lines. (Left panel) Whole cell lysate immunoblots for SHARPIN and GAPDH (loading control). (Right panel) qRT-PCR for relative mRNA levels of SHARPIN. β-Actin was used as an internal control. Values are means ± S.E.M. of three independent experiments. *p < 0.05 from one-way ANOVA test. (**C**) Confirmation of depletion of SHARPIN in lung cancer A549, H460 and SK-MES-1 cell lines. Immunoblots for SHARPIN and GAPDH as control, from indicated cells infected with shRNA targeted against negative control (NC) or against SHARPIN. (**D**–**F**) Cell migration and invasion assays for lung cancer cell line (D) A549, (E) H460 and (F) SK-MES-1 with depletion of GFP (ctr) or SHARPIN. Representative crystal violet staining of invaded cells (upper panel) through a phase-contrast microscope (20X) is shown. Quantification of the invaded cells is shown (down panel). Values are mean ± S.E.M. of three independent experiments. *p < 0.05 from one-way ANOVA test.

To determine the correlation between human lung cancer and SHARPIN expression, RNA-sequencing results from the Cancer Genome Atlas (TCGA) were systematically mined. Interestingly, elevated SHARPIN expression was found in both human lung adenocarcinoma and squamous cell carcinoma compared with that in normal lung samples (Figure 1A). This exciting observation was also supported by results showing that SHARPIN RNA and protein expression is increased in several common lung cancer cell lines compared with those in normal lung cells (Figure 1B). To explore the role of SHARPIN alteration in cancer cells, migration and invasion assays were then performed to characterize changes in the mobility of SHARPIN-depleted cells. Immunoblots for SHARPIN confirmed efficient knockdown of SHARPIN in different lung cancer cells including A549, H460 and SK-MES-1 cells (Figure 1C). Remarkably, cell migration and invasion rates were significantly decreased in A549 (Figure 1D), H460 (Figure 1E) and SK-MES-1 cells (Figure 1F) with the depletion of SHARPIN, compared to those in control cells. These results suggested that SHARPIN plays an essential role in controlling the mobility of lung cancer cells.

### PRMT5 identified as a new cofactor that interacts with SHARPIN

**Figure 2:**
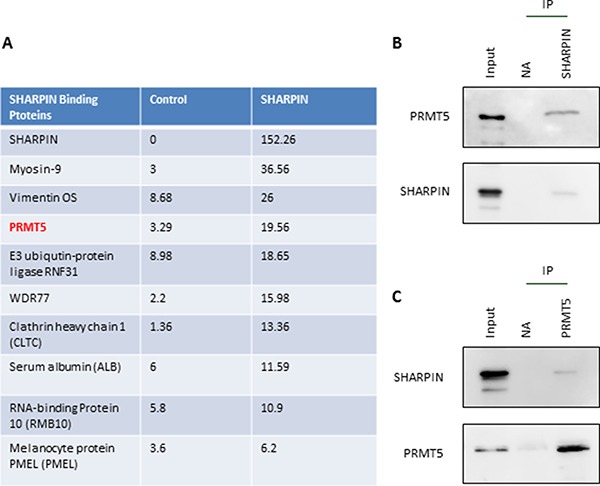
PRMT5 is a new cofactor that interacts with SHARPIN (**A**) LC-MS/MS was performed to identify proteins that interact with SHARPIN in A549 cells. Top 10 functional annotations interacted with SHARPIN were listed. (**B**) SHARPIN Immunoprecipitation (IP) was performed in A549 cell lysates with antibodies directed against SHARPIN or no antibody (NA) as a control. Immunoblots were performed for both PRMT5 and SHARPIN (positive control). (**C**) PRMT5 Immunoprecipitation (IP) was performed in A549 cell lysates with antibodies directed against SHARPIN or no antibody (NA) as a control. Immunoblots were performed for both SHARPIN and PRMT5 (positive control).

To explore the mechanism by which SHARPIN regulates lung cancer cell invasion, we attempted to identify new potential cofactors interacting with SHARPIN. Strikingly, by using LC-MS/MS, we detected several proteins in the SHARPIN immunoprecipitation fractions from A549 cells (Figure 2A). As indicated, a novel histone arginine methyltransferase, PRMT5, was identified as a potential cofactor of SHARPIN. Since PRMT5 is also widely involved in the pathologic progress of cancer metastasis, we tested if PRMT5 was a target of SHARPIN. With immunoprecipitation, we confirmed that SHARPIN interacts with PRMT5. As indicated in Figure 2B, immunoprecipitation was performed with anti-SHARPIN antibody, and immunoblotting with PRMT5 proved the interaction between SHARPIN and PRMT5 (Figure 2B). Conversely, a similar conclusion was drawn from Figure 2C. Thus, we further questioned whether PRMT5 as a novel cofactor of SHARPIN also plays an important role in the invasion of cancer cells.

### PRMT5 mediates invasive phenotype of lung cancer cells

**Figure 3:**
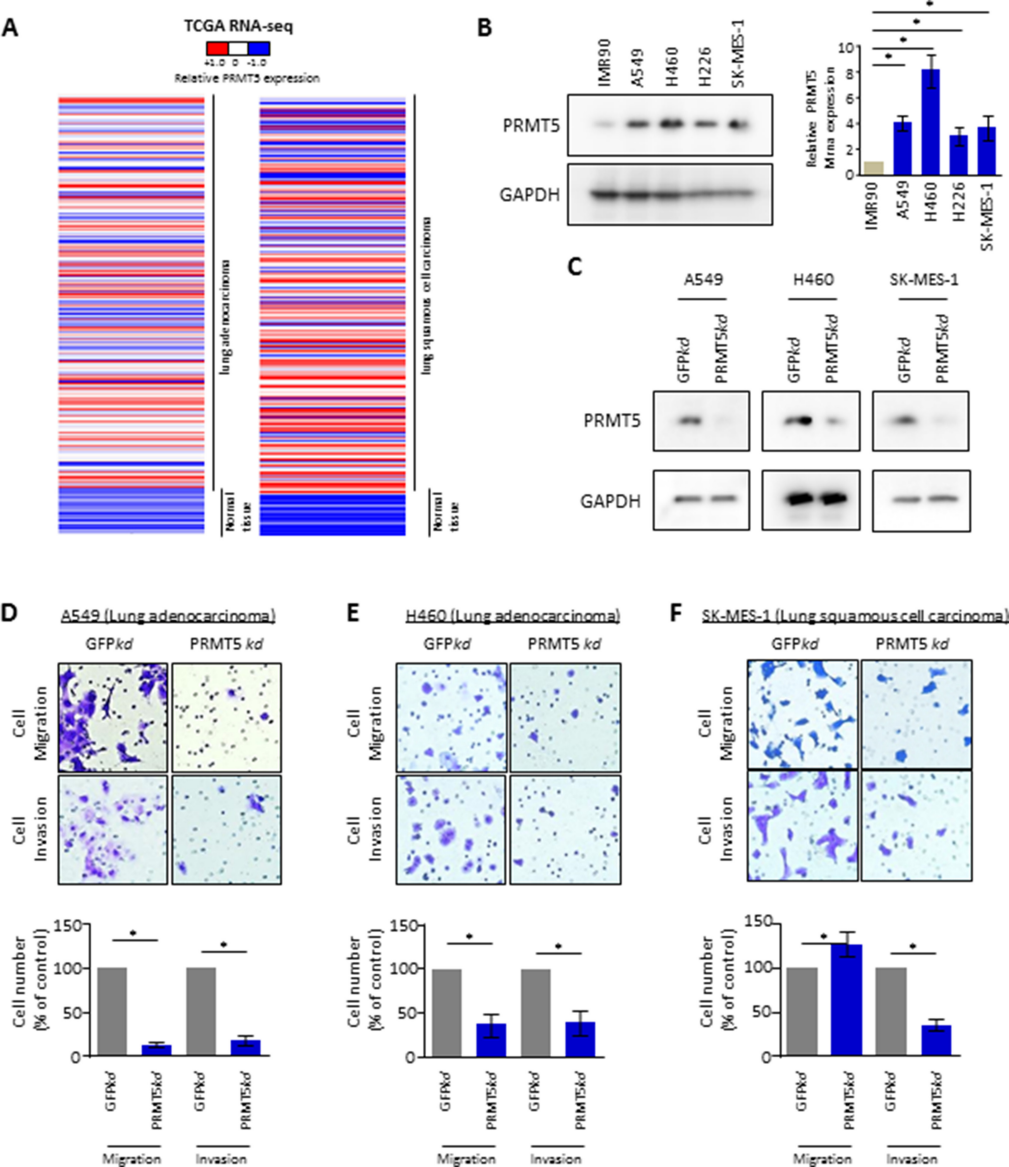
PRMT5 blocked lung cancer cell migration and invasion (**A**) Heat map depicting gene expression of PRMT5 from RNA-Sequencing data with lung adenocarcinoma (LUAD, normal tissues vs tumor tissues) or squamous cell carcinoma (LUSC, normal tissues vs tumor tissues) in The Cancer Genome Atlas (TCGA). Expressions of PRMT5 are ranked by sample types. RNA-Seq values are shown by indicated bar. (**B**) PRMT5 expression profile in lung normal fibroblast cells and lung cancer cell lines. (Left panel) Whole cell lysate immunoblots for PRMT5 and GAPDH (loading control). (Right panel) qRT-PCR for relative mRNA levels of SHARPIN. β-Actin was used as an internal control. Values are means ± S.E.M. of three independent experiments. *p < 0.05 from one-way ANOVA test. (**C**) Confirmation of depletion of PRMT5 in lung cancer A549, H460 and SK-MES-1 cell lines. Immunoblots for PRMT5 and GAPDH as control, from indicated cells infected with shRNA targeted against negative control (NC) or against SHARPIN. (**D**–**F**) Cell migration and invasion assays for lung cancer cell line (D) A549, (E) H460 and (F) SK-MES-1 with depletion of GFP (ctr) or PRMT5. Representative crystal violet staining of invaded cells (upper panel) through a phase-contrast microscope (20X) is shown. Quantification of the invaded cells is shown (down panel). Values are mean ± S.E.M. of three independent experiments. *p < 0.05 from one-way ANOVA test.

Similar to the observations regarding alterations in SHARPIN expression in cancer (Figure 1A), the expression level of PRMT5 was also found to be dramatically elevated in both human lung adenocarcinoma and squamous cell carcinoma compared with that in normal lung samples by analysis of RNA-sequencing from TCGA (Figure 3A). Meanwhile, compared to normal lung cells, lung cancer cells displayed much higher levels of PRMT5 RNA and protein (Figure 3B). shRNA was then used to independently knock down PRMT5 in these lung cancer cell lines (Figure 3C). Knockdown of PRMT5 in A549 (Figure 3D), H460 (Figure 3E) and SK-MES-1 cells (Figure 3F) resulted in significantly reduced rates of migration and invasion compared to those in control cells. These results indicate that PRMT5 is necessary for maintaining malignancy-related cellular properties, including migration and invasion.

### SHARPIN regulates PRMT5-dependent arginine methylation of histones H3 and H4 concomitant with metastasis-related gene expression

**Figure 4:**
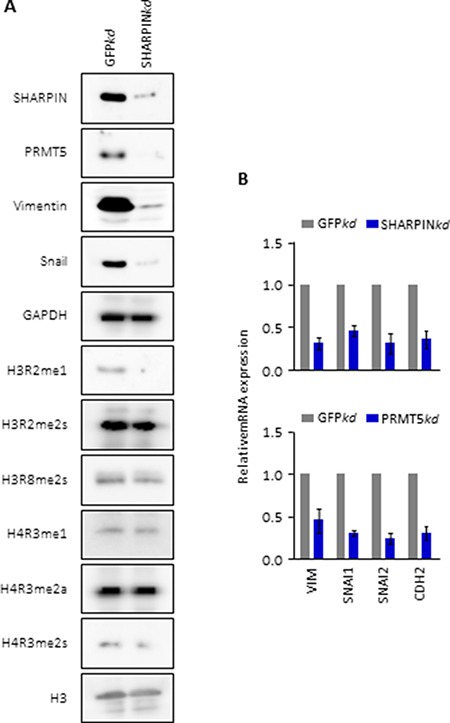
SHARPIN is necessary for PRMT5 and unique histone methylation concomitant with regulation of lung metastasis-related gene expression (**A**) Immunoblots for SHARPIN, PRMT5, metastasis activator genes, and various histone methylarginine PTMs. GAPDH is used as a loading control. The tested A549 cells expressed shRNA targeted against GFP as a control (GFP*kd*) or against SHARPIN (SHARPIN*kd*). (**B**) Relative mRNA levels of indicated metastasis activator genes in A549 cells with depletion of GFP (ctr) vs SHARPIN (upper panel) or PRMT5 (down panel). mRNA was determined by qRT-PCR β-Actin was used as an internal control. Values are means ± S.E.M. of three independent experiments. *p* value is measured with one-way ANOVA test.

We hypothesized that SHARPIN influences local chromatin and gene expression by regulating PTM of PRMT5-enriched histones. We immunoblotted whole cell lysates from SHARPIN-knockdown A549 cells (Figure 4A). Compared with control, SHARPIN knockdown dramatically decreased PRMT5 protein expression. By monitoring the PTM of each histone associated with SHARPIN in SHARPIN-depleted IMR90 cells, we further demonstrated that SHARPIN specifically promotes methylation at H3R2me1 and H4R3me2s (Figure 4A).

Remarkably, SHARPIN knockdown also altered Epithelial-MesenchymalTransition (EMT) protein markers exhibited by the loss of expression of Vimentin and Snail (Figure 4A), consistent with the loss of invasive phenotypes in the SHARPIN-depleted A549 cells (Figure 1D). Candidate metastasis-related genes (VIM, SNAI1, SNAI2 and CDH2) were identified from the literature [14, 15]. By performing qRT-PCR experiments on SHARPIN-depleted A549 cells, we confirmed that the expression of metastasis-related genes is regulated by SHARPIN and PRMT5. As shown in Figure 4B, these genes were significantly reduced in expression after knockdown of either SHARPIN or PRMT5.

Since PRMT5 is also required for mediating the molecular consequences of EMT [14], these observations strongly suggested that SHARPIN is essential for maintaining PRMT5-associated chromatin environments in lung cancer A549 cells, with the activation of expression of cancer metastasis-related genes.

### SHARPIN is essential for PRMT5-mediated H3 arginine methylation in regulating the expression of cancer metastasis-related genes

Since the invasive phenotypes, metastasis-related gene expression and PRMT5-dependent histone PTM of lung cancer cells were directly dependent on SHARPIN-PRMT5, this interaction likely alters the transcriptional outcomes by targeting histones. To test whether SHARPIN maintains the influence of PRMT5 on metastasis-related gene expression by targeting PTM of histones, we performed chromatin immunoprecipitation (ChIP) of promoter elements (-1 kb to +1 kb) of these genes in A549 cell lines depleted of SHARPIN.

**Figure 5:**
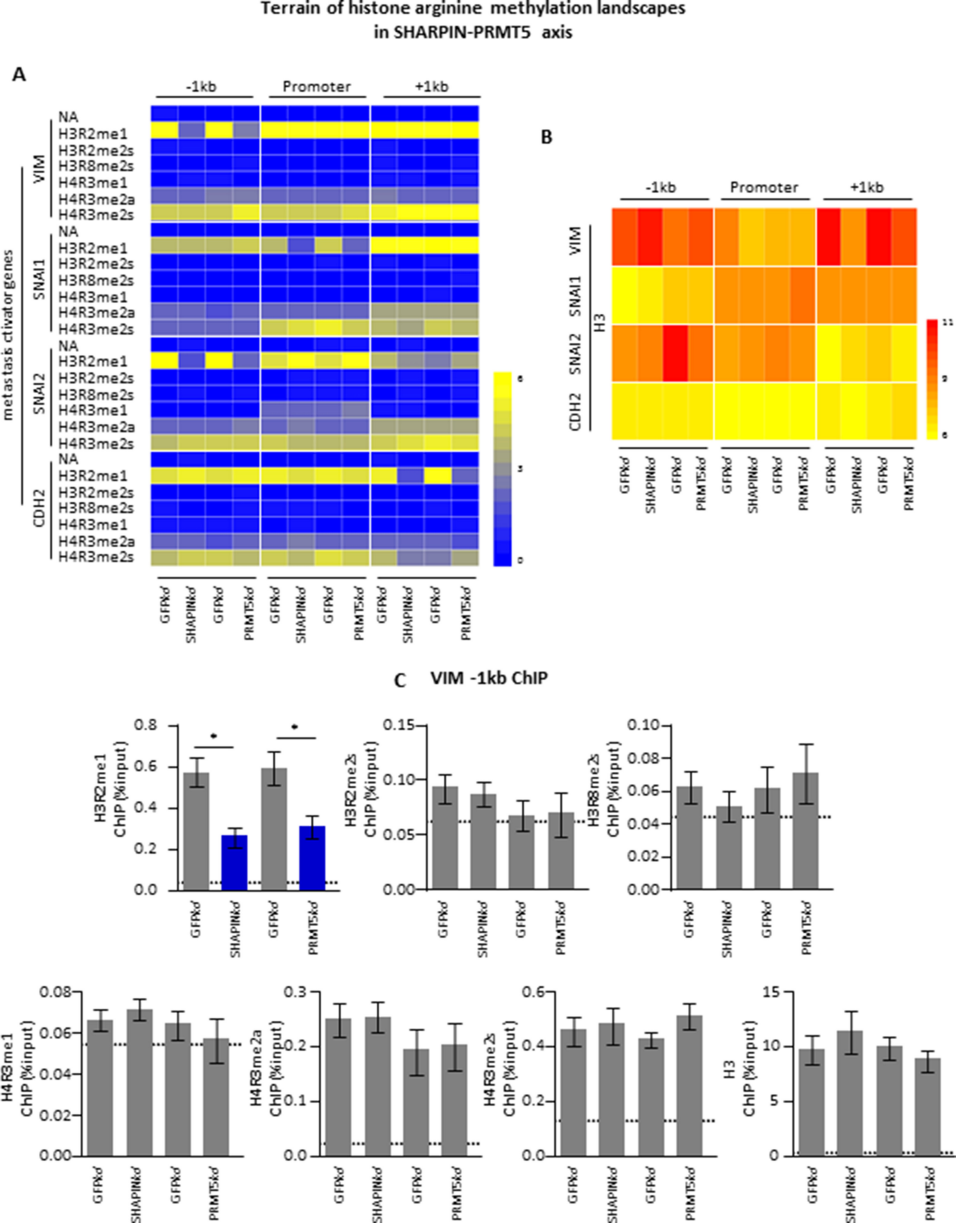
SHARPIN-PRMT5 controls a unique histone methylarginine code to regulate metastasis-related gene expression (**A**) The values of ChIP-qPCR from PRMT5 and various histone methylarginines is used to generated a heatmap to demonstrate the characteristic distribution of histone methylarginines surrounding the candidate genes at -1kb, the promoter, or +1kb regions. The heatmap was arrayed from blue (no enrichment) to yellow (maximal enrichment) as shown by indicated bar. (**B**) Histone H3 is used as control for ChIP experiments (A). Heatmap generated by ChIP-qPCR values for histone H3 values at -1kb, the promoter, or +1kb of the metastasis activator genes. The results indicated the H3 was pulled down evenly. There was no significant H3 enrichment changes between different groups. *p* value is measured with one-way ANOVA test. (**C**) Illustration of ChIP-qPCR results for VIM (-1kb) indicated in (A and B). The horizontal dotted line indicated the upper limit of the 95% confidence interval of the signal from no-antibody (NA) control ChIPs. Values are mean ± S.E.M. of three independent experiments. *P < 0.05 from one-way ANOVA test.

We systematically ChIPed all of the known PRMT5-dependent PTMs including H3R2me1, H3R2me2s, H3R8me2s H4R3me1, and H4R3me2s. The asymmetric mark H4R3me2a was also tested as a negative control for PRMT5 activity (Figure 5A). The control marker H3 was pulled down evenly (Figure 5B). As indicated by the heatmap (Figure 5A), metastasis-related genes were strikingly enriched in H3R2me1, H4R3me2a and H4R3me2s, while the observed enrichment of H3R2me1 was significantly decreased in the SHARPIN-knockdown cells. The ChIP-qPCR profile of all these histone PTMs supports the idea that SHARPIN is essential for PRMT5 in maintaining unique H3 arginine methylation (H3R2me1), which surrounds the promoter regions of cancer metastasis-related genes.

To highlight this novel gene regulation mechanism, we graphed the ChIP-qPCR values for various histone PTMs on VIM (-1 kb) (Figure 5C). As illustrated, among all the tested histone PTMs, H3R2me1, H4R3me2a and H4R3me2s were enriched at the VIM (-1 kb) locus in A549 cells. Surprisingly, with the depletion of SHARPIN, only the distribution of H3R2me1 on this region was significantly decreased.

Taken together, these findings provide insights into the mechanism by which SHARPIN maintains the expression of metastasis-related genes through PRMT5-dependent H3R2me1 arginine methylation of histone H3.

### SHARPIN-PRMT5 promotes a characteristic chromatin environment in metastasis-related genes

**Figure 6:**
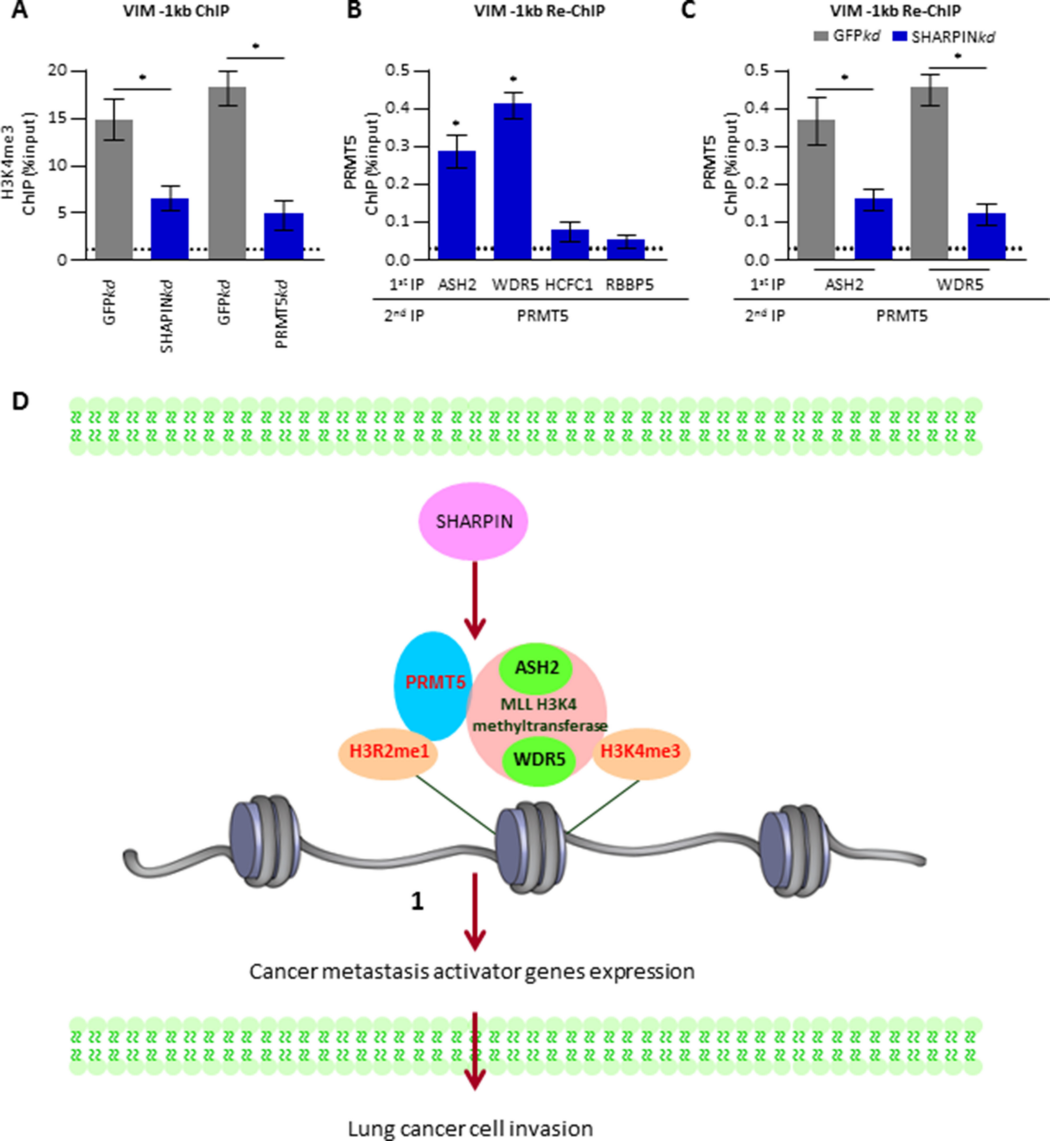
A novel SHARPIN-PRMT5-H3R2me1 axis is involved in regulating of metastasis-related gene expression (**A**) H3K4me3 and H3 (control) ChIP-qPCR experiments of Vim (-1kb) in A549. The tested A549 cells expressed shRNA targeted against GFP as a control (GFP*kd*), against SHARPIN (SHARPIN*kd*) or against PRMT5 (PRMT5*kd*). (**B**) Re-ChIP was performed in A549 cells with 1^st^ round pull down of various MLL complex antibodies including ASH2, WDR5, HCFC1 or RBBP5 as well as 2nd round pull down of PRMT5 antibody. (**C**) A549 cells were depleted of GFP (ctr) or SHARPIN and harvested for Re-ChIP test. Re-ChIP was performed with 1st round pull down of ASH2 or WDR5 antibody as well as 2nd round pull down of PRMT5 antibody. (**D**) Model depicting SHARPIN affecting PRMT5 modulation of metastasis activator gene expression via H3Rme1 histone arginine methylation.

PRMT5-dependent H3R2me1 has been reported to enhance recruitment of the MLL complex for promoting H3K4me3 [14], which acts as a classic gene activating histone marker. We probed the possibility that SHARPIN might be crucial for the PRMT5-mediated epigenetic regulation of gene expression. Using ChIP assay, we confirmed that the knockdown of either SHARPIN or PRMT5 significantly reduced the abundance of H3K4me3 on VIM (-1 kb). With re-ChIP assay, we identified for the first time, the interaction between PRMT5 with all the known components of the MLL complex including ASH2, WDR5, HCFC1 and RBBP5 in A549 cells. The results demonstrated a strong interaction between PRMT5 and ASH2 or WDR5 on the regulatory region (-1 kb) of the VIM gene (Figure 6B). Strikingly, knockdown of SHARPIN dramatically reduced the occupancy of PRMT5 and ASH2 or WDR5 on the same tested region (Figure 6C). These novel findings demonstrate that SHARPIN plays a key role in incorporating PRMT5 into an activating MLL complex (ASH1 and WDR5), which mediates H3K4m3 to regulate the expression of metastasis-related genes.

In a detailed model (Figure 6D), our findings suggest that SHARPIN is responsible for mediating the expression of cancer metastasis-related genes, which leads to changes in the mobility of cancer cells. Meanwhile, PRMT5 is identified as a novel cofactor of SHARPIN, which targets the methylation of histone H3 arginine2 for gene activation through recruitment of the MLL complex (ASH2 and WDR5) to promote H3K4me3.

## DISCUSSION

SHARPIN, a multifunctional molecule, participates in a variety of biological processes, including activation of nuclear factor-κB signaling and inhibition of tumor suppressor genes [3–5]. SHARPIN has been implicated in the pathologic progression in several cancers. Here, we identified that SHARPIN is closely related to lung cancerogenesis. We further showed that SHARPIN plays a novel role in lung cancer metastasis. SHARPIN is mainly localized in the cytoplasm of cancer cells; however, a fraction of SHARPIN is also localized in the nucleus [16, 17], which implies that SHARPIN can interact with specific transcription factors to regulate gene expression. Nevertheless, there stands a long-time puzzle regarding the specific molecule that can integrate SHARPIN signaling with chromatins for the regulation of gene expression. Identification of PRMT5 as a novel cofactor of SHARPIN in our study provided a key to solve this problem.

Although a wealth of literature has supported the essential biological functions of PRMT5 in targeting histones [11, 14, 18], including symmetric dimethylation of histones H2A and H4 on R3 or histone H3 on R8 and asymmetric methylation of H3 on R2, the exact type of histone arginine methylation mediated by SHARPIN-PRMT5 in lung cancer metastasis is still unknown. In this study, we hypothesized that SHARPIN-PRMT5 forms a crucial intermediate in relaying cytoplasmic signaling pathways to chromatins by altering the PTM of histones to control transcription. In this study, we found that SHARPIN activates PRMT5 to specifically target histone H3R2 for monomethylation, which is responsible for the subsequent activation of cancer-related genes.

Histone post-translational modifications (PTMs) are important for proliferation and differentiation of cancer cells, which play a central role in the etiologic and pathologic mechanisms of cancer [19, 20]. Our data indicate that SHARPIN-PRMT5 has distinct roles in orchestrating chromatin environments for cancer-related genes by maintaining the monomethylation of histone H3 arginine and trimethylation of histone lysine4. H3R2me2s has been found to have the capacity to recruit the WDR5 protein, which promotes H3K4me3 to regulate gene expression through its associated MLL lysine methyltransferase [18, 21]. The similarity of the structure of H3R2me1 to that of H3R2me2s suggests that H3R2me1 may also recruit WDR5 through a similar mechanism. In our study, for the first time, we systematically screened the known components of the MLL methyltransferase complex to identify the ones binding to H3R2me1. The exciting results exceeded our expectation, and we speculated that there was a strong interaction between SHARPIN-PRMT5-dependent H3R2me1 and ASH2 or WDR5, which forms the activating MLL complex to mediate H3K4m3 to regulate the expression of metastasis-related genes. These results provide insights into the epigenetic mechanism by which SHARPIN-PRMT5 is involved in cancer metastasis. It is worthwhile to further explore whether this novel epigenetic mechanism is also involved in other types of cancers metastasis.

In conclusion, we demonstrated a new SHARIN-PRMT5-H3R2me1 axis mediating metastasis in invasive lung cancer cells. As new diagnostic molecules and new drug candidates are required for personalized treatment for lung cancer metastasis, it is essential to have more insights into epigenetic gene regulatory pathways. Ubiquitination and methylation are two important post-translational modifications of proteins in human cells, which are widely involved in the spatial and temporal regulation of gene expression [22–26]. Although we have a wealth of knowledge on ubiquitination and methylation as individual modifications, the extent to which the two modifications orchestrate to regulate gene expression in the context of different cellular signaling in cancer cells is poorly understood. Therefore, it is important to determine how the epigenetic landscape is further coordinated by ubiquitination and methylation in future investigations. Meanwhile, more efforts are needed to evaluate and improve the safety of these novel approaches to treat lung cancer metastasis in clinical trials.

## MATERIALS AND METHODS

### Cell culture

IMR90 primary human fetal lung fibroblast cells (ATCC) were cultured in MEM medium with 10% FBS. Various lung cancer cells A549 (ATCC), H460 (ATCC), H226 (ATCC) and SK-MES-1 were cultured in RPMI medium with 10% FBS. Mycoplasma contamination was routinely tested in these cell lines. Stable depletion of SHARPIN, PRMT5 or GFP (control) in A549, H460 and SK-MES-1 were performed by retroviral-mediated expression of shRNA with the pSuper. Retro system obtained from the TRC genome-wide shRNA collection. The SHARPIN shRNA targeting sequence was 5′- GGCUGCAGGUCACACUUGATT-3′. The PRMT5 shRNA targeting sequence was 5′- GCCCA GTTTGAGATGCCTTAT-3′ [14]. The GFP (control) shRNA targeting sequence was 5′-TACAACA GCCACAACGTCTAT-3′ [14]. The cells were selected and maintained under 0.5 mg/ml G418.

### Cell migration and invasion assays

Cell migration and invasion assays were performed in Falcon permeable supports for 24 well plates essentially according to the manufacturer's instruction (Corning, 353097). Cells migrated on the lower surface of the PET membrane were fixed with 4% paraformaldehyde for 15 min at room temperature, then stained with 0.2% crystal violet in 2% ethanol for 20 min at room temperature. The cells were imaged with a Nikon Diaphot phase contrast microscope. At least three separate microscopic fields were counted per membrane.

### Immunoblots and acid-extraction of histones

Whole cell-lysis and acid-extraction of histones were performed as previously described [27]. Briefly, the 90% confluent cells in one 10cm dish were lysed in 100 μl of 0.1% Triton X-100 lysis buffer. After centrifugation at 14,000 r.p.m. for 10 min at 4°C, the supernatant of the lysate was collected as the whole cell-lysis. The pellet was resuspended in 80 μl 0.5 M HCl at 4°C for 2 h under agitation to liberate acid-extractable proteins. After centrifugation at 14,000 r.p.m. for 10 min at 4°C, the supernatant was neutralized with 20 μl 2 M Tris. Immunoblots were performed on PVDF (Immobilon, Millipore) and anti-mouse or anti-rabbit secondary antibody was used for detection by ECL chemiluminescence according to the manufacturer's instructions (Lumigen, TMA-6). Then, immunoblots were imaged with the ImageQuant LAS4000 imaging system. A list of antibodies used is presented in Supplementary Table 1. All immunoblots were repeated at least twice with independent biological samples.

### Co-immunoprecipitation (Co-IP)

Co-IP was performed essentially as previously described [28]. Cell lysates were pre-cleared with protein A-agarose beads at 4°C for 2 h. The resulting supernatant was subsequently incubated at 4°C overnight with protein A-agarose beads reacted with either SHAPRIN rabbit antibody or PRMT5 rabbit antibody, while No-antibody (NA) was used as negative control. The immunoprecipitated proteins were analyzed by subjected to SDS-PAGE and western blotting. All immunoblots have been repeated at least twice with independent biological samples.

### RNA extraction and qRT-PCR

RNA purification and qRT-PCR were performed as described previously [29]. Briefly, mRNA levels were analyzed by reverse transcription followed by quantitative PCR (qRT-PCR). RNA was isolated with Rneasy mini kit (Qiagen, 74104) according to the manufacturer's protocol. The RNA was reverse transcribed with Moloney murine leukemia virus (MMLV) reverse transcriptase (Invitrogen) and a dT18 primer. cDNA, SYBR Green PCR master mix (Roche 04707516001). The efficiency-corrected threshold cycle (ΔCT) method was used to determine the relative levels of RNA. Primer sequences used in this study are listed in Supplementary Table 2.

### Chromatin immunoprecipitation (ChIP) and Re-chromatin immunoprecipitation (Re-ChIP)

Chromatin Immunoprecipitation (ChIP) was performed as previously described [29]. Briefly, cells were grown to 90% confluence in 15 cm dishes, cross-linked with 1% formaldehyde, and sonicated. After sonication, the cross-linked chromatins were immunopreciptated with ChIP-grade antibodies against PRMT5, histone H3 and various histone methylation modification including H3R2me1, H3R2me2s, H3R8me2s, H4R3me1, H4R3me2a and H4R3me2s. Noantibody (NA) controls were always included as negative control. The enrichment of the specific amplified region was detected by quantitative real-time PCR with SYBR Green (Roche 04707516001) and percentage enrichment of each immunoprecipitated material over input chromatin DNA was shown. qRT-PCR with SYBR was performed to detect the indicated regions of the target genes. For Re-Chromatin Immunoprecipitation (Re-ChIP) [30], immunoprecipitated complexes were eluted with the elution buffer (1% SDS, 100mM NaCO3), diluted with the re-ChIP buffer (1% Triton X-100, 2 mM EDTA, 150 mM NaCl, 20 mM Tris pH 8.1), which was subjected to immunoprecipitation with PRMT5 antibody. Antibodies and primers used for ChIP-qPCR are shown in Supplementary Table 1 and Supplementary Table 3 respectively.

### Mass spectrometry

The mass spectrometry was performed essentially as described previously [31]. 10 plates of 15cm dishes of A549 cells were grown to 90% confluence. The cells were collected and lysed in lysis buffer (150 mM NaCl, 50 mM Tris-HCl, pH 7.5, 1% Triton X-100 and and 1× protease inhibitor cocktail). The lysates were precleared with protein A-agarose beads at 4°C for 3 h. The supernatant was used in immunoprecipitations at 4°C overnight with antibody against SHARPIN as indicated. No-antibody (NA) controls were always included. Then, the beads were washed with lysis buffer for 3 times at 4°C. After centrifugation at 4°C, the resulting beads were subjected to trypsin digestion. The supernatant with SHAPIN-binding proteins were detected by mass spectrometry^19^.

### Statistical analysis

All immunoblots were independently performed at least three times. All migration and invasion assays, ChIP, Re-ChIP and RT-qPCR experiments were repeated at least three times with independent biological samples. All data are presented as means ± s.e.m. One-way ANOVA test was used to determine the significance of differences between samples indicated in Figures.

## SUPPLEMENTARY MATERIALS TABLES


