# Supplementary material for: A novel SHARPIN-PRMT5-H3R2me1 axis is essential for lung cancer cell invasion
Source: Oncotarget. 2017 Jul 4;8(33):54809–20. doi: 10.18632/oncotarget.18957 (PMC5589623; doi:10.18632/oncotarget.18957)
Supplement: Supplementary file 1 [file oncotarget-08-54809-s001.pdf]

A novel SHARPIN-PRMT5-H3R2me1 axis is essential for lung cancer cell invasion

SUPPLEMENTARY MATERIALS

Supplementary Table 1: Antibodies used in this study

Factor or PTM	Vendor	Cat Number	Dilution For blotting	Vol.for ChIP (µl)
SHARPIN	Abcam	ab197853	1:5,000	
PRMT5	Millipore	07-405	1:5,000	10
Vimentin	Cell signaling	5741	1:3,000	
Snail	Cell signaling	3879	1:2,000	
GAPDH	Abcam	ab9484	1:1,000	
H3R2me1	Abcam	ab15584	1:5,000	8
H3R2me2s	Abcam	Ab194684	1:5,000	6
H3R8me2s	Novus Biologicals	NB21-1063	1:5,000	10
H4R3me1	Novus Biologicals	NB21-2011	1:10,000	10
H4R3me2a	Active Motif	39705	1:10,000	8
H4R3me2s	Abcam	ab5823	1:10,000	3
H3K4me3	abcam	Ab8580	1:10,000	5
H3	Abcam	ab1791	1:100,000	4
HRP-conjugated anti-rabbit secondary antibody	GE	NA834V	1:30,000	
HRP-conjugated anti-mouse secondary antibody	GE	NA931V	1:30,000	

Supplementary Table 2: RT-qPCR primers used in this study

Gene name	Forward sequence	Reverse sequence
ACTB	AGCTACGAGCTGCCTGAC	AAGGTAGTTTCGTGGATGC
SHARPIN	GCTATTGCAGGTGGAGACGA	GCCTCCTGAAGCTGAACACT
PRMT5	TTGCCGGCTACTTTGAGACT	ACAGATGGTTTGGCCTTCAC
VIM	CCCTCACCTGTGAAGTGGAT	GCTTCAACGGCAAAGTTCTC
CDH1	CAAGTGCCTGCTTTTGATGA	GTTTCTGTGCACACCTGGA
CDH2	TGCTTCAACACGCTTTTGTT	TGGTCTCATCCCCCAAGATA
SNAI1	GCTCCACAAGCACCAAGAGT	ATTCCATGGCAGTGAGAAGG
SNAI2	GAGGAATCTGGCTGCTGTGT	GGTTGGTCAGCACAGGAGAA

Supplementary Table 3: ChIP primers used in this study

Primer set name	Location in the genome	Forward sequence	Reverse sequence
CDH1 −1 kb	chr16:68735940+68736048	TGCCTGGCCCTATTGTTACT	CCCTATGCTGTTGTGGGACT
CDH1 P	chr16:68737215+68737321	GTGAACCCTCAGCCAATCAG	TCACAGGTGCTTTGCAGTTC
CDH1 +1 kb	chr16:68738469+68738561	AGAAATTGCACTCCCACACC	GATCCCCAAATCTGCGTAAA
CDH2 −1 kb	chr18:27950018+27950116	CAGCATGGAGGCACAGTCTA	GAGCTTGCCTCTGGGAATTT
CDH2 P	chr18:27951910+27952010	GGCACATAAAATCCCAGTGC	TGGGCTCAGAGGGAATATCA
CDH2 +1 kb	chr18:27951682+27951787	TGGTCTCATCCCCCAAGATA	TGCTTCAACACGCTTTTGTT
VIM -1 kb	chr10:17226963+17227105	ATGCCTTGTCCTCCTTTTCC	GTGTGCCTGGAACCCTTAGA
VIM P	chr10:17228395+17228494	GGCCCAGCTGTAAGTTGGTA	CCTAGCGGTTTAGGGGAAAC
VIM +1 kb	chr10:17229220+17229290	GAGGGGACCCTCTTTCCTAA	GAGAGTGGCAGAGGACTGGA
SNAI1 −1 kb	chr20:49981560+49981683	CGGCACCAAGTGACTAAACA	CACAGGTCTCACCGTTCTTG
SNAI1 P	chr20:49983118+49983246	GCGAGCTGCAGGACTCTAAT	GTGACTCGATCCTGGCTCA
SNAI1 +1 kb	chr20:49983626+49983698	GGGGTCCTACGTGTGAGAGA	TCCACAGGACAGACCAGGTT
SNAI2 −1 kb	chr8:48921418+48921537	CCTTTACGAACTGAGCCCGT	GTTCACAGCTGTCCCAGAGG
SNAI2 P	chr8:48921190+48921259	TATGTGTGTCCAGTTCGCTGT	CGCTCCTTCCTGGTCAAGAA
SNAI2 +1 kb	chr8:48920720+48920855	CGTCCCCATTGAGGAAGGAG	TTAGAAGGCGGCTTGATGCA
